# Acquisition of multidrug-resistant Enterobacterales during international travel: a systematic review of clinical and microbiological characteristics and meta-analyses of risk factors

**DOI:** 10.1186/s13756-020-00733-6

**Published:** 2020-05-20

**Authors:** Anne F. Voor in ‘t holt, Kees Mourik, Berend Beishuizen, Adriënne S. van der Schoor, Annelies Verbon, Margreet C. Vos, Juliëtte A. Severin

**Affiliations:** 1 Department of Medical Microbiology and Infectious Diseases, Erasmus MC University Medical Centre, Rotterdam, The Netherlands; 2 National Institute for Public Health and the Environment, Bilthoven, The Netherlands

**Keywords:** Travel, Enterobacteriaceae, Enterobacterales, Systematic review, Meta-analysis, Antimicrobial resistance, Epidemiology, Microbiology, Beta-lactamases

## Abstract

**Background:**

International tourism increased from 25 million tourist arrivals in 1950 to over 1.3 billion in 2017. These travelers can be exposed to (multi) resistant microorganisms, may become colonized, and bring them back home. This systematic review aims to identify the carriage rates of multidrug-resistant Enterobacterales (MDR-E) among returning travelers, to identify microbiological methods used, and to identify the leading risk factors for acquiring MDR-E during international travel.

**Methods:**

Articles related to our research question were identified through a literature search in multiple databases (until June 18, 2019) - Embase, Medline Ovid, Cochrane, Scopus, Cinahl, Web of Science, and Google Scholar.

**Results:**

Out of 3211 potentially relevant articles, we included 22 studies in the systematic review, and 12 studies in 7 random-effects meta-analyses. Highest carriage rates of MDR-E were observed after travel to Southern Asia (median 71%), followed by travel to Northern Africa (median 42%). Carbapenemase-producing Enterobacterales (CPE) were identified in 5 out of 22 studies, from a few patients. However, in only eight out of 22 studies (36.4%) the initial laboratory method targeted detection of the presence of CPE in the original samples. The risk factor with the highest pooled odds ratio (OR) for MDR-E was travel to Southern Asia (pooled OR = 14.16, 95% confidence interval [CI] = 5.50 to 36.45), followed by antibiotic use during travel (pooled OR = 2.78, 95% CI = 1.76 to 4.39).

**Conclusions:**

Risk of acquiring MDR-E while travelling increases depending on travel destination and if antibiotics are used during travel. This information is useful for the development of guidelines for healthcare facilities with low MDR-E prevalence rates to prevent admission of carriers without appropriate measures. The impact of such guidelines should be assessed.

## Introduction

Multidrug resistance, defined as acquired non-susceptibility to at least one agent in three or more antimicrobial categories, of clinically important bacteria is recognized as a major threat for human health worldwide. However, remarkable geographical differences in prevalence and trends exist [1, 2]. Multidrug-resistant (MDR) Enterobacterales (MDR-E) that produce extended-spectrum beta-lactamases (ESBL) and/or carbapenemases are of most concern, since these bacteria are able to colonize the human gut and may cause a variety of infections that subsequently require more complicated treatments [3, 4]. Fecal colonization rates with ESBL-producing Enterobacterales (ESBL-E) are estimated to be 14% among healthy individuals worldwide, with an annual increase from 1990 to 2015 of around 5% [5]. These rates are higher in the Mediterranean, the West Pacific, Africa and South-East Asia, and lower in Northern Europe and North America [5]. The carriage rate of carbapenemase-producing Enterobacterales (CPE) in healthy individuals is estimated to be low or absent, although only few studies have included healthy people in a community setting. In East-London, 200 community stool samples were screened and no CPE was identified [6]. CPE are mostly seen in people with exposure to healthcare [7]. In some countries, however, CPE are widespread in the environment [8, 9].

When people travel from low-prevalence areas to areas with a higher prevalence of ESBL-E or CPE in the community, such strains may become part of their gut flora and then carried to the travelers’ home country. This risk is on the rise, since international tourism has increased from 25 million tourist arrivals in 1950, to 1326 million tourist arrivals in 2017 with an expected annual growth of 3.3% [10, 11]. This means that by 2030, 1.8 billion tourist arrivals will be reported. Additionally, between 2015 and 2016, travel to Oceania, Africa and South-East Asia increased the most, by 9.4, 8.1 and 7.8% respectively [10].

Tängdén et al. first reported on travel and acquisition of ESBL-E in 2010 [12]. Since then, numerous reports and several systematic reviews have been published on the relationship between fecal colonization with MDR-E and international travel [13, 14]. However, for healthcare settings, especially those with a low prevalence of MDR-E, it is still unclear how to translate this knowledge into policies or guidelines for infection control and patient care. In addition, it is unclear how travel clinics or general practitioners can use the existing information for pre-travel advice. This review adds to the existing literature by performing an extensive systematic review to describe carriage rates of MDR-E among returning travelers, to describe microbiological methods used, and to perform a meta-analysis in order to identify the leading risk factors for acquiring MDR-E during international travel.

## Methods

This systematic review and meta-analyses followed the guidelines presented in the PRISMA statement (see Additional file 1) [15]. Moreover, this study is an update and extension of the study published by Hassing et al. (Prospero registration number CRD42015024973), whose database search was conducted on August 17, 2015 [16].

### Study selection

Articles related to our research question were identified through a literature search in multiple databases (until June 18, 2019) ─ Embase, Medline Ovid, Cochrane, Scopus, Cinahl, Web of Science, and Google Scholar (see Additional file 2). The search was not limited by language, date of publication, country of publication or study design.

We used the following inclusion criteria during the study selection: (i) related to foreign travel, (ii) reports on systematic and selective screening for the carriage of ESBL-E and/or CPE among travelers without signs of infections when performing the screening, and (iii) report on fecal Enterobacterales carriage. We excluded studies related to nonhuman infections, hospital studies, studies about symptomatic patients (e.g., travelers’ diarrhea [TD]), conference abstracts, letters to the editor, commentaries, weekly reports, and editorials. First, titles and abstracts of all retrieved citations were screened independently by KM and AFV. After this screening, KM, AFV and BB performed a second screening based on the full-text. Disagreements were resolved by discussion. Reference lists of reviews and systematic reviews on the same subject, which were identified during the literature search, were screened to identify additional studies that had been missed by our search strategy.

### Data extraction

We designed a data extraction form and pilot-tested it on two randomly selected articles, and redefined it according to the outcomes. The following data were extracted by AFV and BB: first author, journal, year published, country, study design, study period, where were the participants recruited (e.g. travel agency, vaccination clinic), total number of participants, mean age, percentage female, mean duration of travel, sample method, microorganism(s) studied, co-travelers or household members included, percentage of carriage before and after travel, acquisition rate, acquisition rate to household members, acquisition rates for each United Nations geographical region, laboratory methods (e.g. species determination, phenotypic approaches, molecular approaches), risk factors and protective factors identified in multivariable models; and corresponding odds ratio, 95% confidence interval and *P*-value. The completed data extraction form was sent to the corresponding author of the original manuscript to verify the extracted data, and to gain additional information if relevant. In case we did not receive any response after the given deadline (i.e. 2 weeks), a reminder was sent. If no response was received and crucial information was missing, the study was excluded.

### Data analysis

#### Carriage rates of multidrug-resistant Enterobacterales by travel destination

The following geographical classification was used, based on the United Nations geographical regions: (i) Southern Asia, (ii) Asia except Southern Asia, (iii) Northern Africa, (iv) Sub-Saharan Africa, (v) South and Central America, (vi) North America, (vii) Europe, (viii) Oceania (see Additional file 3). Carriage rates immediately after return were grouped into the following 5 categories, and for a visual presentation of the results a color was added from green to dark red: (i) 0–20%, low, green; (ii) 21–40%, moderate, yellow; (iii) 41–60%, high, orange; (iv) 61–80%, very high, red; (v) 81–100%, extremely high, dark red.

### Meta-analysis

All risk factors extracted from the articles for which an odds ratio (OR) with 95% confidence interval (95%CI) was reported were grouped into eight categories: (i) diarrhea during travel, (ii) antibiotic use during travel, (iii) travel to Southern Asia, (iv) behavior during travel, (v) food consumption during travel, (vi) length of stay, (vii) sex, and (viii) age.

The meta-analyses for each category were performed using StatsDirect statistical software (Altrincham, United Kingdom) including the random-effects model of DerSimonian and Laird [17]. We used a random-effects model to limit the influence of heterogeneity. A *P*-value of < 0.05 was considered statistically significant. Publication bias was examined visually with use of funnel plots, and assessed with the Egger and Begg-Mazumdar indicators [18, 19].

### Study quality

The methodological quality was assessed for all included studies using the strengthening the reporting of observational studies in epidemiology (STROBE) guideline [20]. Studies with a score ≤ 15 out of 33 points were considered to be of relatively low methodological quality, studies receiving a quality score of 16–19 points were considered as of moderate quality, and studies with ≥20 points were considered to have a relatively high study quality. Study quality was not considered an exclusion criterion.

## Results

**Fig. 1:**
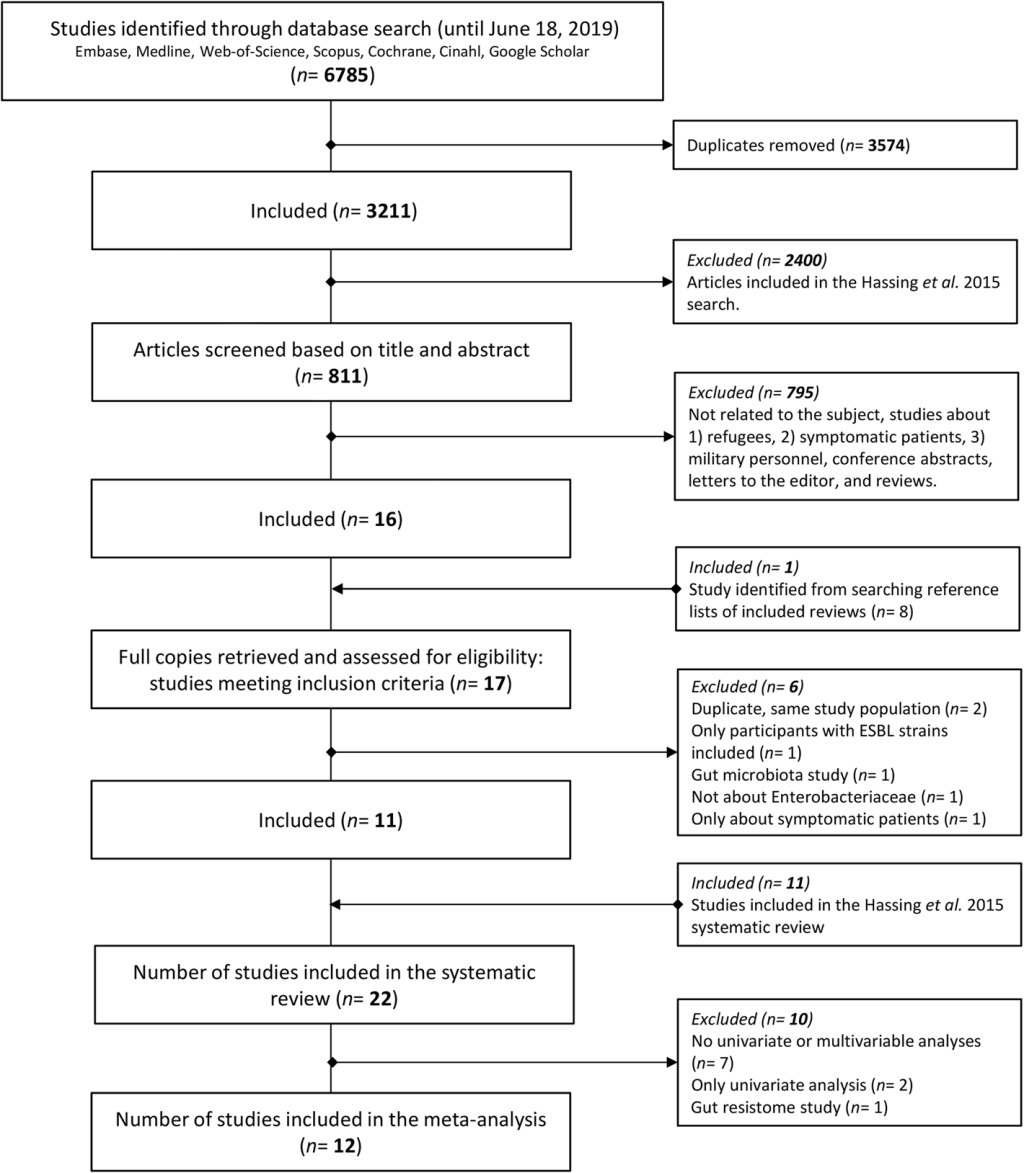
Flow diagram of study selection

The literature search identified 3211 non-duplicate articles, of which 811 articles were potentially relevant for this current study (Fig. 1). Titles and abstracts of these 811 articles were screened, which resulted in the exclusion of 795 articles (98%). One additional study was included after searching the reference lists of reviews of interest. The remaining 17 articles underwent a second screening based on the full-text, after which 6 articles were excluded (35.3%) (Fig. 1). The remaining 11 studies were added to the 11 previously identified by Hassing et al., and used in his review. Hence, in total 22 articles were included in this systematic review [16]. Of the 22 included articles, 12 were included in random-effects meta-analyses (Fig. 1).

For the 22 included studies, the corresponding author was contacted to provide feedback on extracted data or to request additional information. The corresponding authors of all studies (100%) responded to our request to provide feedback on the extracted data. For three studies, we requested additional information on the multivariable analysis since crucial information was missing. Unfortunately, additional information was not received and these studies were therefore excluded from the random-effects meta-analyses.

### Study characteristics

All 22 included studies were prospective cohort studies. The studies were conducted in Western Europe (*n* = 17, 77.3%), North America (*n* = 2, 9.1%), Japan (*n* = 2, 9.1%), and Australia (*n* = 1, 4.5%). The characteristics of the studies are shown in Table 1. Fifteen studies investigated travelers visiting a travel or vaccination clinic, one study investigated hospital staff and contacts, one study investigated healthcare students, one study investigated business travelers, one study investigated Hajj pilgrims, and two studies did not state the type of study population. The studies by van Hattem et al. and Arcilla et al. both investigated participants enrolled in the COMBAT-study (ClinicalTrials.gov identifier: NCT01676974); van Hattem et al. reported on CPE acquisition, and Arcilla et al. on ESBL-E acquisition [36, 37].

**Table 1:** Study characteristics of the 22 included studies

Study	Year	Country	Study period	Population characteristic	Study size^**a**^	Proportion of MDR ***E. coli***^***e***^ in post-travel isolates	Sample time (range) before/after travel	Median duration of travel in days (range)	Follow-up of carriage
Kennedy [21]	2010	Australia	January 2008–April 2009	Hospital staff and contacts	102	> 92% *E. coli*^b^	Within 2 weeks before and after	21 (9–135)	6 months
Tängdén [12]	2010	Sweden	November 2007–January 2009	Travel clinic	100	100% *E. coli* (24/24^b^)	Unknown	14 (7–182)	6 months
Weisenberg [22]	2012	United States	July 2009–February 2010	Travel clinic	28	*E. coli* 100% (7/7^b^)	1 week before/within 1 week after	14 (8–42)	None
Östholm-Balkhed* [23, 24]	2013	Sweden	September 2008–April 2009	Vaccination clinic	231	90% *E. coli* (104/116)^b^	15 (1–114) days/ 3 (0–191) days	16 (4–119)	12 months
Paltansing* [25]	2013	The Netherlands	March 2011–September 2011	Travel clinic	370	92% *E. coli* (146/158)^c^	Immediately before and after	21 (6–90)	6 months
Kuenzli* [26]	2014	Switzerland	December 2012–October 2013	Travel clinic	190	98% *E. coli* (157/161^b^)	Week before/directly after	Mean; 18 (5–35)	None
von Wintersdorff [27]	2014	The Netherlands	November 2010–August 2012	Travel clinic	122	ND	Before and immediately after	21 (5–240)	None
Angelin* [28]	2015	Sweden	April 2010–January 2014	Healthcare students	99	100% *E. coli* (35/35^c^)	Close to departure/ 1–2 weeks after return	45 (13–365)	None
Kantele* [29]	2015	Finland	March 2009–February 2010	Travel clinic	430	97% *E. coli* (94/97^b^)	Before/first (or second) stool after	Mean; 19 (4–133)	12 months
Lübbert [30]	2015	Germany	May 2013–April 2014	Travel clinic	205	92% *E. coli* (58/63^b^)	Before/within 1 week after	21 (2–218)	6 months
Ruppé* [31]	2015	France	February 2012–April 2013	Vaccination centers	574	93% *E. coli* (491/526^b^)	Within 1 week before and after	20 (IQR 15–30)	12 months
Bernasconi [32]	2016	Switzerland	January 2015–August 2015	Unknown	38	90% *E. coli* (26/29^b^)	Within 1 week before and after	Mean; 15 (8–35)	6 months
Mizuno [33]	2016	Japan	September 2012–March 2015	Business travelers	57	ND	Before and at time of return	> 6 months	None
Reuland* [34]	2016	The Netherlands	April 2012–April 2013	Vaccination clinic	445	97% *E. coli* (95/98^b^)	Before/within 2 weeks after	Mean; 14 (1–105)	None
Vading* [35]	2016	Sweden	April 2013–May 2015	Travel clinic	188	97% *E. coli* (65/67^b^)	Unknown	14 (IQR 8–20)	10 to 26 months
van Hattem^d^ [36]	2016	The Netherlands	November 2012–November 2013	Travel clinic	2001	60% *E. coli* (3/5^b^)	Before/immediately and 1 month after travel	20 (IQR 15–25)	12 months
Arcilla*^d^ [37]	2017	The Netherlands	November 2012–November 2013	Travel clinic	2001	88% *E. coli* (759/859^b^)	Before/immediately and 1 month after travel	20 (IQR 15–25)	12 months
Leangapichart* [38]	2017	France	Hajj 2013 & 2014	Hajj pilgrims	218	ND	Just before departure and after the Hajj just before return	22 and 24	None
Peirano* [39]	2017	Canada	January 2012–July 2014	Travel clinic	116	100% *E. coli* (124/124^b^)	Before /within 1 week after	10–38	6 months
Bevan [40]	2018	United Kingdom	March 2015–June 2016	University and university hospital	18	100% *E.* coli (16/16)	As close to the time of sample submission and after	21, mean 27	Up to 12 months
Nakayama [41]	2018	Japan	June 2015–August 2016	Unknown	19	100% *E. coli*	Before and up to 2 weeks after	2–12 days	None
Schaumburg* [42]	2019	Germany/the Netherlands	October 2016–March 2018	Vaccination center	132	ESBL-producing Enterobacterales	Up to 1 week before departure, during travel and up to 1 week after return	Mean: 18.7, maximum of six weeks	6 months (137–420 days after return)

Abbreviations: *E. coli*, *Escherichia coli*; *MDR* Multidrug-resistant; *ND* No data; *, included in the meta-analyses

^a^ Number of travelers who provided pre-travel and post-travel samples

^b^ MDR microorganisms newly acquired during travel

^c^ Data about post-travel samples

^d^ Reported on the same study population, however, van Hattem et al. reported on CPE acquisition, and Arcilla et al. on ESBL-E acquisition

^e^ Including ESBL-producing *E. coli* and carbapenemase-producing *E. coli*

The median study size was 160 participants, ranging from 18 to 2001 participants, and the median age of participants ranged from 25 years to 66 years, with participants included from 0 to 84 years old. The median proportion of women was 61%, ranging from 26 to 78%; one study did not report the gender of the participants. Most studies had participants with a similar median duration of travel, ranging from median 14 to 21 days. However, the study investigating healthcare students had a median duration of travel of 45 days (13–365) [28] and in the study investigating business travelers, participants travelled for at least 6 months [33]. Sample collection in the included studies was via stool sample (*n* = 14, 63.6%), rectal swab (*n* = 6, 27.3%), a rectal swab or a stool sample (*n* = 1, 4.5%), or a rectal or perianal swab (*n* = 1, 4.5%). Four studies also investigated co-travelers in addition to the study population [12, 29, 36, 37]. Three studies did not report on identification of microorganisms, and one study investigated a variety of antimicrobial-resistant bacteria: ESBL-E, CPE and colistin-resistant Gram-negative bacteria. Between the latter two groups, *Enterobacter cloacae* and *Escherichia coli* were the most frequently identified Enterobacterales, respectively. In all other studies, ESBL-positive *E. coli* was the most dominant MDR-E identified in post-travel samples.

Only one study clearly defined TD as more than 3 loose/liquid stools per 24 h or more frequently than normal for an individual [29] and one study referred to the World Health Organization (WHO) definition [42]. The other included studies just asked in questionnaires if the participant had experienced TD yes or no.

### Microbiological methods

Enrichment was used in 12 out of 22 included studies (54.5%), all with a different composition, and 21 out of 22 included studies (95.5%) used selective agar plates, with 13 studies using ChromID ESBL (Table 2). The method most often used for antimicrobial susceptibility testing was the VITEK2 system (bioMérieux, 10 out of 22, 45.5%), and phenotypic confirmation of ESBL-production was most often performed by disk-diffusion (12 out of 22, 54.5%) (Table 2).

**Table 2:** Microbiological methods of the 22 included studies

Study	Enrichment		Selective media		AST	Confirmation of ESBL	CPE-targeted isolation method	CPE screening in isolates
Kennedy [21]	Yes	BHI broth with vancomycin disk	Yes	MacConkey with NAL disk, horse BA with gentamicin, ChromID ESBL	VITEK2	Disk-diffusion, PCR for *bla*_TEM_, *bla*_SHV_, and *bla*_CTX-M_	No	No
Tängdén [12]	Yes	LB broth with cefotaxime	Yes	MacConkey with cefotaxime and ceftazidime disks	E-test	Disk-diffusion	No	No, only carbapenem AST
Weisenberg [22]	No	NA	Yes	MacConkey with cefpodoxime	VITEK2	Disk-diffusion, PCR for *bla*_TEM_, *bla*_SHV_, and *bla*_CTX-M_	No	Yes, PCR for *bla*_KPC_, *bla*_IMP_, *bla*_VIM_, *bla*_OXA-48_, *bla*_NDM_
Östholm-Balkhed [23]	No	NA	Yes	ChromID ESBL, chromogenic UTI agar with antibiotic disks	E-test	E-test, PCR for *bla*_TEM_, *bla*_SHV_, and *bla*_CTX-M_	No	No, only carbapenem AST
Paltansing [25]	Yes	TSB with cefotaxime and vancomycin	Yes	ChromID ESBL	VITEK2	Disk-diffusion, microarray for *bla*_TEM_, *bla*_SHV_, and *bla*_CTX-M_	No	Yes, microarray to detect *bla*_KPC_, *bla*_IMP_, *bla*_VIM_, *bla*_OXA-48_, *bla*_NDM-1_
Kuenzli [26]	Yes	TSB with 0.5% sodium chloride	Yes	ChromID ESBL, MacConkey with ertapenem disk	VITEK2	Disk-diffusion, selection of isolates: microarray for *bla*_TEM_, *bla*_SHV_, and *bla*_CTX-M_	Yes	Yes, modified Hodge, selection of isolates: microarray for *bla*_KPC_, *bla*_IMP_, *bla*_VIM_, *bla*_OXA-48_, *bla*_NDM_
von Wintersdorff [27]	NA	NA	NA	NA	NA	PCR for *bla*_CTX-M_	No	Yes, PCR for *bla*_NDM_
Angelin [28]	No	NA	Yes	ChromID ESBL	Disk-diffusion	E-test	No	Yes, disk-diffusion for *bla*_OXA-48_ and *bla*_OXA-181_ and CT103XL microarray
Kantele [29]	No	NA	Yes	ESBL, KPC (CHROMagar)	VITEK2	Disk-diffusion	Yes	No, only AST
Lübbert [30]	No	NA	Yes	CHROMagar ESBL, CHROMagar KPC plate	Microbroth dilution	E-test, PCR for *bla*_TEM_, *bla*_SHV_, and *bla*_CTX-M_	Yes	Yes, multiplex PCR for *bla*_KPC_, *bla*_IMP_, *bla*_VIM_, *bla*_OXA-48_, *bla*_NDM_
Ruppé [31]	Yes	(1) BHI broth with cefotaxime; (2) BHI broth with ertapenem	Yes	(1) With and without enrichment: ChromID ESBL agar; without enrichment: bi-valve ESBL agar; (2) Drigalski agar with ertapenem and imipenem E-test	Disk-diffusion	PCR for *bla*_TEM_, *bla*_SHV_, *bla*_CTX-M_, and *bla*_VEB_	Yes	Yes, PCR for *bla*_KPC_, *bla*_IMP_, *bla*_VIM_, *bla*_OXA-48_, *bla*_NDM_
Bernasconi [32]	Yes	LB broth with a cefuroxime disk	Yes	BLSE, ChromID ESBL, Supercarba selective plates	Microdilution	CT103XL microarray	No	Yes, CT103XL microarray
Mizuno [33]	No	NA	Yes	ChromID ESBL	MicroScan Neg Combo 6.11 J panel	Disk-diffusion	No	No, only imipenem AST
Reuland [34]	Yes	TSB with ampicillin	Yes	EbSA ESBL agar, CLED agar with ciprofloxacin disk	VITEK2	Disk-diffusion, PCR for ESBL genes	No	Yes, ertapenem E-test, PCR for carbapenemase genes followed by sequencing
Vading [35]	Yes	LB broth with meropenem	Yes	In-house chromogenic base with cloxacillin and meropenem; without enrichment: ChromID ESBL	Disk-diffusion	Vitek2, Check-MDR microarray	Yes	Yes, Check-MDR microarray
van Hattem [36]	Yes	TSB with vancomycin	Yes	ChromID ESBL, chromID OXA-48 agar	VITEK2, E-test	Disk-diffusion, Identibac® AMR08 microarray	Yes	Yes, Identibac® AMR08 microarray and targeted PCR and DNA sequencing
Arcilla [37]	Yes	TSB with vancomycin	Yes	ChromID ESBL	VITEK2	Disk-diffusion	No	No
Leangapichart [38, 43, 44]	Yes	TSB	Yes	MacConkey with cefotaxime and Cepacia agar	Disk-diffusion	PCR for *bla*_TEM_, *bla*_SHV_, and *bla*_CTX-M_	No	No, only imipenem AST
Peirano [39]	No	NA	Yes	ChromID ESBL, chromID-CARBA SMART	VITEK2	Disk-diffusion, PCR for *bla*_TEM_, *bla*_SHV_, and *bla*_CTX-M_	Yes	Partly, carbapenem AST, PCR for *bla*_OXA_
Bevan [40]	Yes	BHI broth with cefpodoxime disk	Yes	Oxoid ESBL brilliance agar, Oxoid UTI brilliance agar with cefpodoxime disk	NP	PCR for CTX-M ESBL genes	No	Yes, WGS and bioinformatics screening
Nakayama [41]	No	NA	Yes	CHROMagar ECC with 1 μg/mL cefotaxime	Disk diffusion	Double-disk synergy test, PCR for ESBL genes	No	No, only meropenem AST
Schaumburg [42]	No	NA	Yes	ChromID-ESBL, chromID-CARBA	VITEK2	Double-disk diffusion	Yes	Yes, modified Hodge test and PCR for *bla*_KPC2–15_, *bla*_VIM1–37_, *bla*_NDM1–7_, *bla*_OXA-48_, *bla*_OXA-181_

Abbreviations: *NAL* Nalidixic acid; *NA* Not applicable; *NR* Not reported; *AST* Antimicrobial susceptibility testing; *TSB* Tryptic soy broth; *ESBL* Extended-spectrum beta-lactamase; *KPC Klebsiella pneumoniae* carbapenemase; *BA* Blood agar; *LB* Luria-Bertani; *BHI* Brain heart infusion; *CLED* Cystine lactose electrolyte-deficient medium; *SMART* Solutions to manage the antimicrobial resistance threat; *NP* Not performed; *WGS* Whole-genome sequencing

In eight out of 22 studies (36.4%) the initial laboratory method targeted detection of the presence of CPE in the original samples, i.e. a selective agar plate method without a pre-enrichment with a broth containing second or third generation cephalosporins (Table 2) [26, 29–31, 35, 36, 39, 42]. In only 4 of these eight studies, a CPE was found [26, 31, 36, 42]. In 13 out of the 22 studies (59.1%), screening for CPE was carried out in isolates or directly on the specimen by a variety of phenotypic and genotypic methods (Table 2). In other studies, carbapenem susceptibility testing was partly or not performed, or isolates with reduced susceptibility to carbapenems were not further analyzed. Therefore, in those studies CPE could have remained unidentified.

### Carriage of multidrug-resistant Enterobacterales

#### International travelers

Table 3 shows the rates of travelers acquiring CPE, or MDR-E (i.e. without CPE) during international travel. It was not possible to report ESBL-E separate from MDR-E, since multiple studies, when reporting prevalence rates, combined ESBL-E and for example AmpC-producing *Enterobacterales*. Highest carriage rates of MDR-E were observed after travel to Southern Asia (Table 3), with proportions ranging from 29 to 88%, with as median 71%. Second was travel to Northern Africa, with proportions ranging from 31 to 100%, with as median 42%. CPE carriage was only identified in 5 studies [26, 31, 34, 36, 42], and mainly found in *E. coli*. Carbapenemases identified were IMI-2, NDM, NDM-1, NDM-1/2, NDM-7, OXA-48, OXA-181 and OXA-244.

**Table 3:**
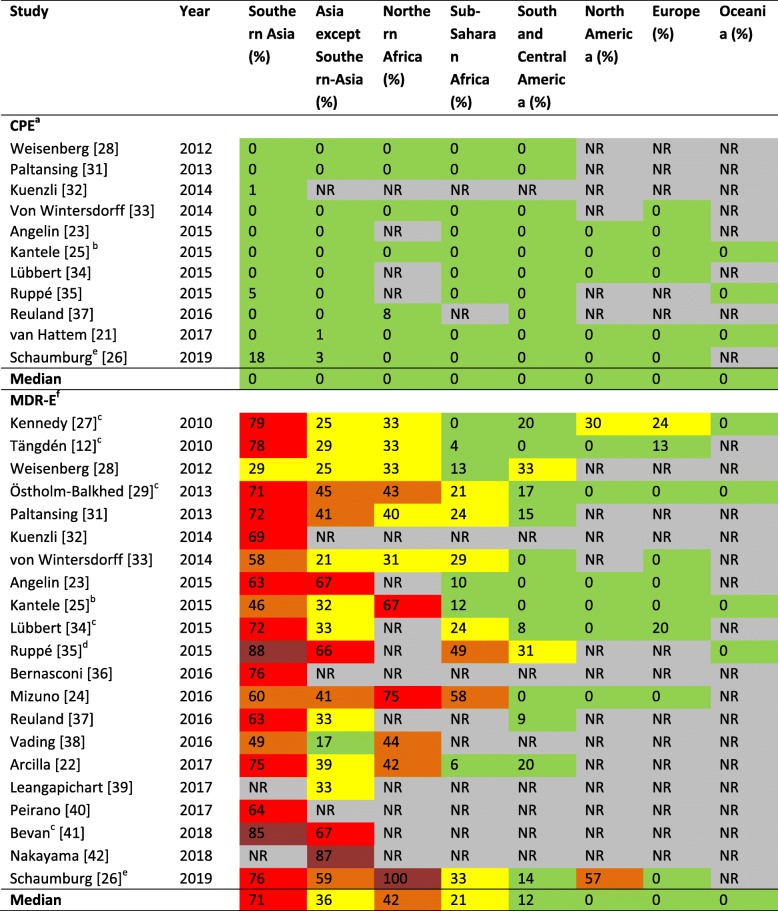
Proportion of travelers who acquired a resistant microorganism after international travel

Abbreviations: *NR* Not reported; *CPE* Carbapenemase-producing *Enterobacterales*; *MDR-E* Multidrug-resistant *Enterobacterales*

Colors: (i) 0–20%, low, green; (ii) 21–40%, moderate, yellow; (iii) 41–60%, high, orange; (iv) 61–80%, very high, red; (v) 81–100%, extremely high, dark red.

^a^ Only noted for studies that used methods to be able to identify CPE as described in Table 2.

^b^ Travelers who visited several regions are arranged to the region in which they spend the most time.

^c^ Travelers who visited several regions are arranged to all of the visited regions.

^d^ 42 travelers visited several countries in Asia and may therefore be arranged in several columns in the table; 28 of them acquired a MDR-E.

^e^ Carriage rates after travel from travelers to Southern-Asia (CPE: 3 out of 17, ESBL: 13 out of 17), Asia except Southern Asia (CPE: 1 out of 29, ESBL: 17 out of 29), Northern Africa (ESBL: 3 out of 3) and Sub-Saharan Africa (ESBL: 9 out of 27) were received from the corresponding author.

^f^ Not including CPE. It was not possible to report ESBL-E separate from MDR-E.

### Household members

Acquisition of MDR-E from the traveler to a non-travelling household member was described by 3 studies [25, 36, 37]. In the study by Paltansing et al., 1 out of 11 (9.1%) household members carried the same ESBL-producing *E. coli* as the traveler [25]. In the study by Arcilla et al., 13 out of 168 (7.7%) household members carried a microorganism with the same ESBL group as the traveler [37]. When the authors estimated the transmission rate after introduction into a household using a Markov model, the probability of transmission was 12% (95% CI = 5 to 18%) [37]. In the study by van Hattem et al., acquisition of a *bla*_OXA-244_-positive *E. coli* from a traveler to a household member was highly suspected. Three months after travel a *bla*_OXA-244_-positive *E. coli* with a similar AFLP pattern was isolated from a fecal sample from a spouse and travel companion. All other fecal specimens from this household member were CPE negative, which suggested post-travel acquisition of the same bacterium [36].

### Persistence of colonization and subsequent infections

Fourteen studies performed follow-up analysis of persistence of MDR-E colonization [12, 21, 24, 25, 29–32, 35–37, 39, 40, 42], ranging from 6 to 26 months (Table 1). The median reported persistence rate of acquired MDR-E at one, three, six and 12 months after return was 42.9, 22.4, 18.0 and 4.2% respectively. Arcilla et al. also reported on the rate of intermittent carriage of acquired MDR-E, which was 2.6% of all follow-up participants at 3 months, 3.0% at 6 months and 4.7% at 12 months [37]. Schaumburg et al. also calculated the median time of event-free survival for ESBL-E (i.e. time to first colonization with ESBL-E during travel), which was 8 days [42].

Five studies [12, 21, 30, 31, 35] investigated whether the colonizing pathogen caused clinical infection afterwards. Only a few clinical infections resulting from colonization with MDR-E have been reported. The study by Kennedy et al. reported one urinary tract infection (UTI) out of 50 follow-up participants caused by an *E. coli* with the same resistance pattern as the colonizing *E. coli* [21]. Ruppé et al. reported that eight out of 245 follow-up participants had contracted a UTI during the follow-up period, but no microbiological data was available to confirm that these infections were caused by the colonizing pathogen [31]. Studies by Lübbert et al., Tängdén et al. and Vading et al. reported no clinical infections in respectively 58, 21, and 56 follow-up participants [12, 30, 35].

### Protective factors and risk factors

We identified 12 studies describing protective and/or risk factors for acquiring MDR-E during travel, obtained from multivariable analyses (see Additional file 4). The highest OR was reported for the risk factor TD (OR = 31.00, 95% CI = 2.70 to 358.10). Examples of identified protective factors were handwashing with soap before meals, a beach holiday, tap water consumption, and travel to various countries compared to traveling to Asia (see Additional file 4).

All identified risk factors, protective factors, and factors identified as non-significant in multivariable models were grouped into 8 categories. For these 8 categories, 7 meta-analyses were performed. For the category length of stay, three factors were identified. However, for one factor the confidence interval was missing. Therefore, for the category length of stay no meta-analysis was performed. The factor antibiotic use during travel was most frequently described (*n* = 13 times identified).

**Fig. 2:**
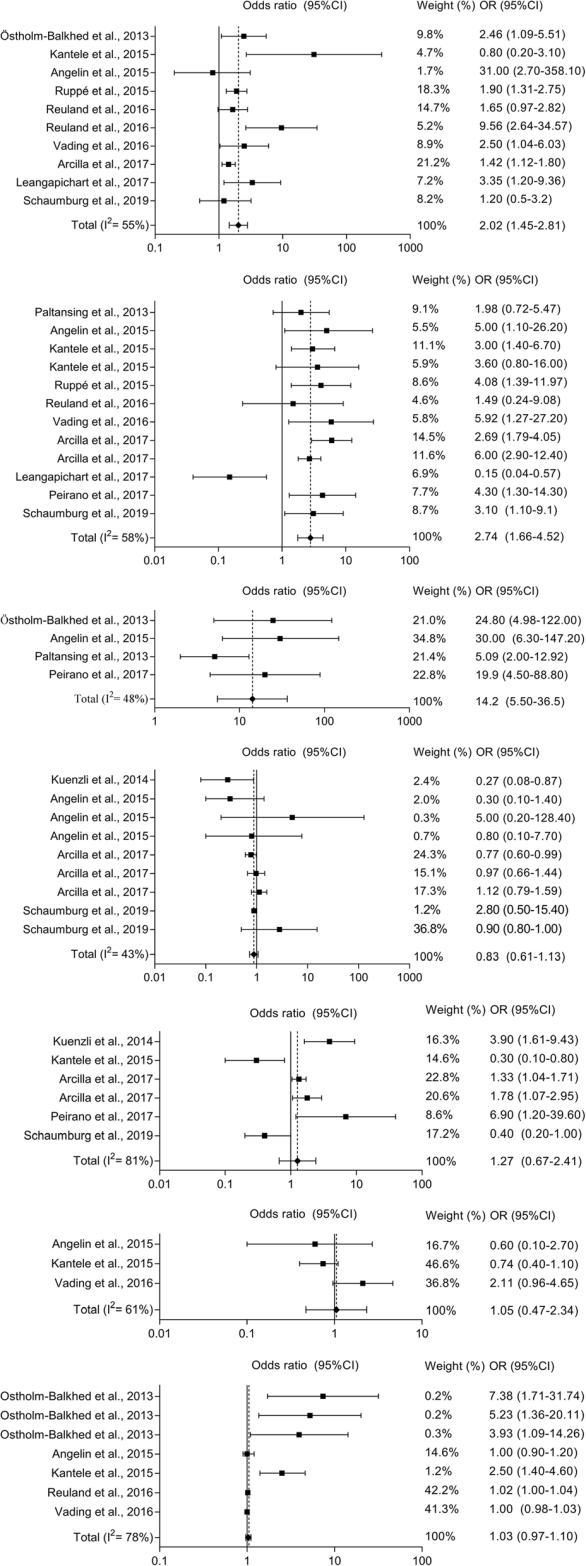
Forest plots of random-effects meta-analyses of risk factors for acquiring multidrug-resistant Enterobacterales during international travel (**a** to **g** appear from top to bottom). (**a**) Experienced diarrhea while travelling (i.e. TD); (**b**) antibiotic use during travel; **c**) travelled to Southern Asia; (**d**) behavior during travel (e.g. brought disposable gloves, consumed bottled water); (**e**) food consumption during travel (e.g. ice cream and pastry consumption, meals at street food stalls); (**f**) male gender; (**g**) older age

The risk factor with highest pooled OR was found to be travel to Southern Asia (pooled OR = 14.16, 95% CI = 5.50 to 36.45) (Fig. 2-c), followed by antibiotic use during travel (pooled OR = 2.78, 95% CI = 1.76 to 4.39) (Fig. 2-b) and TD (pooled OR = 2.02, 95% CI = 1.45 to 2.81) (Fig. 2-a). The factors behavior during travel (Fig. 2-d), food consumption during travel (Fig. 2-e), male gender (Fig. 2-f), and older age (Fig. 2-g) were identified as non-significant factors. Publication bias indicators Begg-Mazumdar (Kendall’s tau) and Egger both showed a statistically significant result in the following meta-analyses: travel to Southern Asia, and older age (see Additional file 5). Funnel plots are available in Additional file 5.

### Study quality

A quality assessment was performed for all included studies; *n* = 22. Overall, the studies scored between 9 and 26 out of 33 points, with a median of 18 points. Six studies had a low methodological quality [22, 27, 28, 32, 33, 38, 41], seven a moderate methodological quality [12, 21, 23, 25, 26, 30, 36], and six studies a high methodological quality [29, 31, 34, 35, 37, 39, 40, 42].

## Discussion

### Summary of evidence

We identified that when travelling to Southern Asia (i.e. Afghanistan, Bangladesh, Bhutan, India, Iran, Maldives, Nepal, Pakistan and Sri Lanka) people are at highest risk of acquiring and carrying a MDR-E upon return. Though, acquisition of MDR-E when visiting Northern Africa or Asia except Southern Asia was also high (Table 3). Additionally, we showed that acquiring a CPE while travelling is still rare, which is supported by the findings of Jans et al. [45]. However, it should be emphasized that in most studies a culture method was used that was not specifically targeting CPE. Especially CPE with OXA-48-like carbapenemases may be missed [36]. The risk factors for acquiring MDR-E in order of those with the highest to those with the lowest pooled OR are: (i) travel to Southern Asia; (ii) antibiotic use during travel; and (iii) TD. Older age, sex, food consumption during travel and behavior during travel were found to be non-significant (Fig. 2). With this systematic review, we aimed to provide aggregated data on acquisition of MDR-E and risk factors for MDR-E acquisition during international travel, which can be useful for the development of guidelines and policies in areas with a low prevalence of MDR-E.

Travel to Asia, especially to India, is a known high risk for acquiring MDR-E. MDR-E are highly prevalent in this area because of the overuse of antibiotics, the lack of (clean) toilets and the lack of clean water. Hereby, bacteria can become resistant and are easily spread between people and to the environment. TD is associated with contaminated food or water, and is related to the lack of hygiene and sanitation [46]. Bacteria are responsible for the majority of cases [46]. TD in combination with antibiotic use does not only increase the risk of acquiring MDR-E, but also selects for antibiotic resistant bacteria [46–49]. Our results in combination with the studies by Kantele et al. highlight the need to avoid antibiotic use in mild to moderate TD [47–49]. Because most diarrheal episodes are self-limiting, it is only important to avoid dehydration [46]. Additionally, only one study used a clear definition of TD [29]. As described by Lääveri et al., the impact of the definition of TD is substantial on the results and conclusions [50].

Interestingly, food consumption – a known risk factor for TD and thus acquiring MDR-E – was identified as non-significant. It may be that, because of all warnings and available guides, people are aware of the risks and stopped eating food from street vendors, raw food, and stopped drinking tap water, milk from open containers and fountain drinks [51]. Alternatively, it is also possible that recall bias played a role in the questionnaires’ outcomes, and food consumption was rarely identified as a risk factor because people unknowingly eat risky food. In our opinion, this is more likely, as it may be difficult for travelers to determine if establishments adhere to food safety standards [52, 53].

### Towards a guideline

Although in our opinion the aggregated data do support the implementation of additional recommendations that can be given by travel clinics and general practitioners to people before travelling, there are still a number of knowledge gaps that need be filled before national and international guidelines on infection control (screening and/or isolation) and patient care (adjustment of empiric treatment) for healthcare facilities can be developed. First, the proportion of people with recent travel history to a foreign country with increased risk of MDR-E acquisition among patients admitted to hospitals is currently unknown. Second, it is unknown whether the strains that are carried by travelers do spread in hospitals, although it is known that in general ESBL-E and CPE can be transmitted between patients and into the hospital environment, especially when contact precautions are not taken, which can lead to outbreaks [54]. The fact that not only strains, but also resistance genes on mobile genetic elements such as plasmids can spread, makes this knowledge gap even more difficult to resolve. The cost-effectiveness of a program that would include screening and subsequent isolation of recent travelers can therefore not be estimated with the currently available data, nor can the overall impact of such a program on healthcare workers, laboratories and patients. The threshold of a carriage rate after travel that warrants screening and/or isolation is also an unresolved issue, but is likely to be dependent on the local carriage rates. For example, when travelling to Sub-Saharan Africa, 17% of travelers acquire ESBL-E. For the Netherlands, a country with a carriage rate in the community of 5.3 to 9.9%, 17% can be considered as high [54]. However, for example in countries with higher community carriage rates, other approaches may be more applicable. Such policies would also require systematic surveillance of carriage rates amongst travelers, or of local carriage rates. The burden of disease of travel-related MDR-E is also unknown. Follow-up data on infections in travelers is scarce, as is data on phylogenetic groups (PG) of *E. coli* and virulence factors in general. The limited available data suggest that infections are rare, clones may belong to low-virulent sequence types, and the PG varies between studies [12, 25, 26, 30, 35, 40, 55]. Third, most studies were performed in Europe and included travelers who visited a travel clinic. Therefore, just a few studies included travelers who visited Europe. In addition, few travelers to North America or Oceania were included in the studies in this review, possibly due to travelers not seeing a travel clinic when visiting these continents. Travelers visiting friends and relatives abroad are also underrepresented, since they usually do not seek health advice in a travel clinic before travelling. Additional studies are needed to assess the risks of these groups of travelers.

### Strengths and limitations

A strength of our study is that it is an extensive literature search. In addition to the systematic review by Hassing et al., we performed meta-analyses to identify the main risk factors, looked more into detail to the laboratory methods and subsequent possibility to identify CPE and identified knowledge gaps. In addition, we performed an in-depth analysis about carriage rates (e.g. carriage rates of travelers, acquisition to household members and persistence of carriage).

This study has some limitations. First, the heterogeneity of included studies. We included studies performed in different countries and studying different types and groups of travelers. Additionally, the prevalence of MDR-E in each country is different and this was not incorporated in the risk factor analysis. To limit the influence of heterogeneity, in the meta-analysis we used a random effects model. Second, publication bias was present in several meta-analyses. Despite our extensive search for all available evidence, small studies with no effect are simply not performed and/or published. However, because of our extensive search, we think that the influence of publication bias to our results and conclusions is limited. Third, seven studies were included with a low methodological quality, of which 2 were included in the meta-analyses. These were relatively small studies, which did not have a big influence in weight on the pooled estimate. Therefore, we consider the influence of studies with a low methodological quality as limited.

## Conclusion

This systematic review shows that travel to South Asia, together with antibiotic use and TD, are leading risk factors for acquiring MDR-E. It is advisable for travelers to contact a travel clinic in their home country before travel to be informed about TD and antibiotic use, and to limit self-prescribing of antibiotics and buying antibiotics over the counter during travel when suffering from TD. Acquisition of CPE during travel is still rare, but possibly underreported. The information in this review is useful for the development of guidelines for healthcare facilities with low MDR-E prevalence rates to prevent admission of potential carriers of MDR-E without appropriate measures. However, we identified a number of knowledge gaps that should be filled in before guidelines for healthcare facilities can be developed and implemented, since the impact of the measures cannot be estimated yet.

### COVID-19

Currently, we are in the midst of the COVID-19 pandemic. Governments are discouraging or forbidding travel of any kind, and calling on everyone to stay at home as much as possible. Additionally, several countries have implemented a full lockdown or shelter-in-place measures. Healthcare systems are severely affected. Furthermore, these measures have a significant impact on domestic and international travel. This also affects the spread of MDR-E: we expect a decreased transmission rate during this period due to the decrease in (inter)national travel. However, an increased use of antibiotics has also been observed. We expect that this, combined with overcrowding and a shortage of personal protective equipment in hospitals, will lead to an increase of local spread of MDR-E, and consequently, we expect an increased local prevalence of MDR-E in low-and-middle income countries and in Southern European countries. If in the second half of 2020 international travel is resumed due to relaxing of COVID-19 measures, we will see the results of this local spread. We expect that the proportion of travelers who acquire a resistant microorganism after international travel will increase after COVID-19. Future surveys will provide more insight in the effect of the COVID-19 pandemic on travel-related spread of MDR-E.

## Supplementary information

**Additional file 1:.** text file: The PRISMA Checklist.

**Additional file 2:.** text file: Literature search strategy – list of search terms.

**Additional file 3:.** text file: Geographical regions and countries.

**Additional file 4:.** text file: Reported risk factors and protective factors.

**Additional file 5:.** text file: Risk of publication bias – funnel plots.

## Data Availability

All data generated or analyzed during this study are included in this published article and its additional files.

## Abbreviations

ESBL: Extended-spectrum beta-lactamase
ESBL-E: ESBL-producing Enterobacterales
CPE: Carbapenemase-producing Enterobacterales
MDR-E: Multidrug-resistant Enterobacterales
OR: Odds ratio
95% CI: 95% Confidence interval
STROBE: Strengthening the reporting of observational studies in epidemiology guideline
TD: Travelers’ diarrhea
PG: Phylogenetic groups
