# Supplementary material for: Acquisition of multidrug-resistant Enterobacterales during international travel: a systematic review of clinical and microbiological characteristics and meta-analyses of risk factors
Source: Antimicrob Resist Infect Control. 2020 May 20;9:71. doi: 10.1186/s13756-020-00733-6 (PMC7237615; doi:10.1186/s13756-020-00733-6)
Supplement: Supplementary file 2 — Additional file 2:. text file: Literature search strategy – list of search terms. [file 13756_2020_733_MOESM2_ESM.docx]

**Additional file 2. Literature search strategy - list of search terms**

**Embase.com**

('Gram negative bacterium'/exp OR 'Gram negative infection'/de OR Enterobacteriaceae/de OR Escherichia/exp OR Klebsiella/exp OR Salmonella/exp OR Shigella/exp OR Yersinia/exp OR 'Enterobacteriaceae infection'/exp OR ('Gram negative' OR Enterobacteri* OR (Enter* NEXT/1 bacteria*) OR Enterobacter* OR Escherichia* OR 'e coli' OR Klebsiella* OR Salmonell* OR Shigell* OR Yersinia*):ab,ti) AND (travel/de OR 'traveller diarrhea'/de OR aviation/exp OR (travel* OR touris* OR turista OR aviation OR 'air transport' OR airport*):ab,ti) AND ('antibiotic resistance'/exp OR 'multidrug resistance'/de OR 'drug resistance'/de OR 'antibiotic sensitivity'/de OR 'bacterial colonization'/exp OR 'bacterium carrier'/de OR (resistan* OR coloni* OR ((antibiotic* OR antimicrob*) NEAR/3 sensitivit*) OR susceptib* OR carriage* OR carrier*):ab,ti) NOT ([animals]/lim NOT [humans]/lim)

**Medline Ovid**

(exp "Gram-Negative Bacteria"/ OR exp "Gram-Negative Bacterial Infections"/ OR Enterobacteriaceae/ OR exp Escherichia/ OR exp Klebsiella/ OR exp Salmonella/ OR exp Shigella/ OR exp Yersinia/ OR exp "Enterobacteriaceae infections"/ OR ("Gram negative" OR Enterobacteri* OR (Enter* ADJ bacteria*) OR Enterobacter* OR Escherichia* OR "e coli" OR Klebsiella* OR Salmonell* OR Shigell* OR Yersinia*).ab,ti.) AND (travel/ OR "Travel Medicine"/ OR exp aviation/ OR (travel* OR touris* OR turista OR aviation OR "air transport" OR airport*).ab,ti.) AND (exp "Drug Resistance, Microbial"/ OR exp "Drug Resistance, Multiple"/ OR "drug resistance"/ OR "bacterium carrier"/ OR (resistan* OR coloni* OR ((antibiotic* OR antimicrob*) ADJ3 sensitivit*) OR susceptib* OR carriage* OR carrier*).ab,ti.) NOT (exp animals/ NOT humans/)

**Cochrane CENTRAL**

(('Gram negative' OR Enterobacteri* OR (Enter* NEXT/1 bacteria*) OR Enterobacter* OR Escherichia* OR 'e coli' OR Klebsiella* OR Salmonell* OR Shigell* OR Yersinia*):ab,ti) AND ((travel* OR touris* OR turista OR aviation OR 'air transport' OR airport*):ab,ti) AND ((resistan* OR coloni* OR ((antibiotic* OR antimicrob*) NEAR/3 sensitivit*) OR susceptib* OR carriage* OR carrier*):ab,ti)

**Web-of-science**

TS=((("Gram negative" OR Enterobacteri* OR (Enter* NEAR/1 bacteria*) OR Enterobacter* OR Escherichia* OR "e coli" OR Klebsiella* OR Salmonell* OR Shigell* OR Yersinia*)) AND ((travel* OR touris* OR turista OR aviation OR "air transport" OR airport*)) AND ((resistan* OR coloni* OR ((antibiotic* OR antimicrob*) NEAR/3 sensitivit*) OR susceptib* OR carriage* OR carrier*)))

**Scopus**

TITLE-ABS-KEY((("Gram negative" OR Enterobacteri* OR (Enter* W/1 bacteria*) OR Enterobacter* OR Escherichia* OR "e coli" OR Klebsiella* OR Salmonell* OR Shigell* OR Yersinia*)) AND ((travel* OR touris* OR turista OR aviation OR "air transport" OR airport*)) AND ((resistan* OR coloni* OR ((antibiotic* OR antimicrob*) W/3 sensitivit*) OR susceptib* OR carriage* OR carrier*)))

**Cinahl**

(MH "Gram-Negative Bacteria+" OR MH "Gram-Negative Bacterial Infections+" OR MH Enterobacteriaceae+ OR MH "Enterobacteriaceae Infections+"OR MH Escherichia+ OR MH Yersinia+ OR TI ("Gram negative" OR Enterobacteri* OR (Enter* N bacteria*) OR Enterobacter* OR Escherichia* OR "e coli" OR Klebsiella* OR Salmonell* OR Shigell* OR Yersinia*) OR AB ("Gram negative" OR Enterobacteri* OR (Enter* N bacteria*) OR Enterobacter* OR Escherichia* OR "e coli" OR Klebsiella* OR Salmonell* OR Shigell* OR Yersinia*)) AND (travel+ OR "Travel Medicine+" OR MH aviation+ OR TI (travel* OR touris* OR turista OR aviation OR "air transport" OR airport*) OR AB (travel* OR touris* OR turista OR aviation OR "air transport" OR airport*)) AND (MH "Drug Resistance, Microbial+" OR MH "drug resistance+" OR TI (resistan* OR coloni* OR ((antibiotic* OR antimicrob*) N3 sensitivit*) OR susceptib* OR carriage* OR carrier*) OR AB (resistan* OR coloni* OR ((antibiotic* OR antimicrob*) N3 sensitivit*) OR susceptib* OR carriage* OR carrier*)) NOT (MH animals+ NOT humans+)

**Google Scholar**

"Gram negative"|Enterobacteriaceae|Escherichia|Klebsiella|Salmonella|Shigella|Yersinia travel|traveller|tourist|tourism resistance|resistant|colonization|colonisation|susceptibility|carriage|carrier
