# Supplementary material for: Acquisition of multidrug-resistant Enterobacterales during international travel: a systematic review of clinical and microbiological characteristics and meta-analyses of risk factors
Source: Antimicrob Resist Infect Control. 2020 May 20;9:71. doi: 10.1186/s13756-020-00733-6 (PMC7237615; doi:10.1186/s13756-020-00733-6)
Supplement: Supplementary file 3 — Additional file 3:. text file: Geographical regions and countries. [file 13756_2020_733_MOESM3_ESM.docx]

**Additional file 3. Geographical regions and countries**

| **Region** | **Countries (and autonomous territories)** |
| --- | --- |
| Southern Asia | Afghanistan, Bangladesh, Bhutan, India, Iran, Maldives, Nepal, Pakistan, Sri Lanka. |
| Asia except Southern Asia | Armenia, Azerbaijan, Bahrain, Brunei, Cambodia, China, Cyprus, Georgia, Hong Kong, Indonesia, Iraq, Israel, Jordan, Japan, Kazakhstan, Kuwait, Kyrgyzstan, Laos, Lebanon, Mongolia, Malaysia, Myanmar, North Korea, Oman, Philippines, Qatar, Saudi Arabia, South Korea, Singapore, Palestine, Syria, Taiwan, Tajikistan, Thailand, Timor-Leste, Turkey, Turkmenistan, United Arab Emirates, Uzbekistan, Vietnam, Yemen. |
| Northern Africa | Algeria, Egypt, Libya, Morocco, Sudan, Tunisia, Western Sahara. |
| Sub-Saharan Africa | Angola, Benin, Botswana, Burkina Faso, Burundi, Cameroon, Cape Verde, Central African Republic, Chad, Comoros, Congo (Brazzaville), Côte d’Ivoire, Democratic Republic of the Congo, Djibouti, Equatorial Guinea, Eritrea, Ethiopia, Gabon, Ghana, Guinea, Guinea-Bissau, Kenya, Lesotho, Liberia, Madagascar, Malawi, Mali, Mauritania, Mauritius, Mozambique, Namibia, Niger, Nigeria, Réunion, Rwanda, São Tomé and Príncipe, Senegal, Seychelles, Sierra Leone, Somalia, South Africa, Sudan, Swaziland, Tanzania, The Gambia, Togo, Uganda, Zambia, Zimbabwe. |
| South and Central America | Anguilla, Antigua and Barbuda, Argentina, Aruba, Bahamas, Barbados, Belize, Bolivia, Bonaire, Sint Eustatius and Saba, Brazil, British Virgin Islands, Cayman Islands, Chile, Colombia, Costa Rica, Cuba, Curaçao, Dominica, Dominican Republic, Ecuador, El Salvador, Falkland Islands, French Guiana, Grenada, Guadeloupe, Guatemala, Guyana, Haiti, Honduras, Jamaica, Martinique, Mexico, Montserrat, Nicaragua, Panama, Paraguay, Peru, Puerto Rico, Saint Kitts and Nevis, Saint Lucia, Saint Martin, Saint Vincent and the Grenadines, Saint-Barthélemy, Sint Maarten, Suriname, Trinidad and Tobago, Turks and Caicos Islands, US Virgin Islands, Uruguay, Venezuela. |
| North America | Bermuda, Canada, Greenland, Saint Pierre and Miquelon, United States. |
| Europe | Åland Islands, Albania, Andorra, Austria, Belarus, Belgium, Bosnia and Herzegovina, Bulgaria, Channel Islands, Croatia, Czech Republic, Denmark, Estonia, Faeroe Islands, Finland, the former Yugoslav Republic of Macedonia, France, Germany, Gibraltar, Greece, the Holy See, Hungary, Iceland, Ireland, Isle of Man, Italy, Latvia, Liechtenstein, Lithuania, Luxembourg, Malta, Monaco, Montenegro, Netherlands, Norway, Poland, Portugal, Moldova, Romania, Russia, San Marino, Serbia, Slovakia, Slovenia, Spain, Svalbard and Jan Mayen, Sweden, Switzerland, Ukraine, United Kingdom. |
| Oceania | American Samoa, Australia, Cook Islands, Fiji, French Polynesia, Guam, Kiribati, Marshall Islands, Micronesia, Nauru, New Caledonia, New Zealand, Niue, Norfolk Island, Northern Mariana Islands, Palau, Papua New Guinea, Pitcairn Islands, Samoa, Solomon Islands, Tokelau, Tonga, Tuvalu, Vanuatu, Wallis and Futuna. |
