# Supplementary material for: Acquisition of multidrug-resistant Enterobacterales during international travel: a systematic review of clinical and microbiological characteristics and meta-analyses of risk factors
Source: Antimicrob Resist Infect Control. 2020 May 20;9:71. doi: 10.1186/s13756-020-00733-6 (PMC7237615; doi:10.1186/s13756-020-00733-6)
Supplement: Supplementary file 4 — Additional file 4:. text file: Reported risk factors and protective factors. [file 13756_2020_733_MOESM4_ESM.docx]

**Additional file 4. Reported risk factors and protective factors**

| **No.** | **First author** | **Year** | **Factor** | **Meta-analyses** | **RF/PF** | **Travelers total** | **With MO** | **WithoutMO** | **PE** | **PE2** | **L 95%CI** | **U 95%CI** | **P-value** |  |
| --- | --- | --- | --- | --- | --- | --- | --- | --- | --- | --- | --- | --- | --- | --- |
| 1 | Östholm-Balkhed | 2013 | Travel to Africa, north of equator | excl. not possible to group | RF | 226 | 68 | 158 | OR | 4.94 | 1.8 | 13.6 | 0.002 |  |
| 2 | Östholm-Balkhed | 2013 | Travel to Asia (except Indian subcontinent) | excl. not possible to group | RF | 226 | 68 | 158 | OR | 8.63 | 3.42 | 21.7 | <0.001 |  |
| 3 | Östholm-Balkhed | 2013 | Travel to Indian subcontinent | 3. Travel to southern-Asia | RF | 226 | 68 | 158 | OR | 24.8 | 4.98 | 122 | <0.001 |  |
| 4 | Östholm-Balkhed | 2013 | Travel in previous 5 years | excl. not possible to group | NS | 226 | 68 | 158 | OR | 4.44 | 0.99 | 20 | 0.052 |  |
| 5 | Östholm-Balkhed | 2013 | hospital care in Sweden in previous 5 years | excl. not possible to group | NS | 226 | 68 | 158 | OR | 0.49 | 0.21 | 1.12 | 0.093 |  |
| 6 | Östholm-Balkhed | 2013 | backpacker-style journey | excl. not possible to group | NS | 226 | 68 | 158 | OR | 3.04 | 0.7 | 13.2 | 0.136 |  |
| 7 | Östholm-Balkhed | 2013 | Diarrhoea during journey | 1. Travelers’ diarrhoea | RF | 226 | 68 | 158 | OR | 2.46 | 1.09 | 5.51 | 0.029 |  |
| 8 | Östholm-Balkhed | 2013 | Other gastrointestinal symptoms during journey | excl. not possible to group | RF | 226 | 68 | 158 | OR | 2.99 | 1.04 | 8.51 | 0.041 |  |
| 9 | Östholm-Balkhed | 2013 | Fever | excl. not possible to group | PF | 226 | 68 | 158 | OR | 0.2 | 0.04 | 0.96 | 0.044 |  |
| 10 | Östholm-Balkhed | 2013 | ≥65y compared to 18-34y of age | 8. Age | RF | 226 | 68 | 158 | OR | 7.38 | 1.71 | 31.74 | 0.007 |  |
| 11 | Östholm-Balkhed | 2013 | 35-49y compared to 18-34y of age | 8. Age | RF | 226 | 68 | 158 | OR | 5.23 | 1.36 | 20.11 | 0.016 |  |
| 12 | Östholm-Balkhed | 2013 | 50-64y compared to 18-34y of age | 8. Age | RF | 226 | 68 | 158 | OR | 3.93 | 1.09 | 14.26 | 0.037 |  |
| 13 | Kantele | 2015 | Meals with locals | 5. Food consumption during travel | PF | 430 | 90 | 340 | OR | 0.3 | 0.1 | 0.8 | 0.01 |  |
| 14 | Kantele | 2015 | antimicrobial use for travelers’ diarrhoea | 2. Antibiotic use during travel | RF | 430 | 90 | 340 | OR | 3 | 1.4 | 6.7 | 0.01 |  |
| 15 | Kantele | 2015 | Travelers diarrhoea | 1. Travelers’ diarrhoea | RF | 430 | 90 | 340 | OR | 31 | 2.7 | 358.1 | 0.01 |  |
| 16 | Kantele | 2015 | Travel to sub-Saharan Africa compared to South Asia | excl. comparison | PF | 430 | 90 | 340 | OR | 0.1 | 0.1 | 0.3 | <0.01 |  |
| 17 | Kantele | 2015 | Contact with local healthcare | excl. not possible to group | NS | 430 | 90 | 340 | OR | 2.1 | 0.8 | 5.6 | 0.12 |  |
| 18 | Kantele | 2015 | AB use for other indications than TD | 2. Antibiotic use during travel | NS | 430 | 90 | 340 | OR | 3.6 | 0.8 | 16 | 0.1 |  |
| 19 | Kantele | 2015 | Travel to north-Africa & middle east compared to south Asia | excl. comparison | NS | 430 | 90 | 340 | OR | 1 | 0.2 | 1.2 | 0.95 |  |
| 20 | Kantele | 2015 | travel to east Asia compared to south Asia | excl. comparison | NS | 430 | 90 | 340 | OR | 0.6 | 0.1 | 5.1 | 0.67 |  |
| 21 | Kantele | 2015 | travel to southeast Asia compared to south Asia | excl. comparison | NS | 430 | 90 | 340 | OR | 0.6 | 0.3 | 1.3 | 0.24 |  |
| 22 | Kantele | 2015 | Gender | 7. Gender | NS | 430 | 90 | 340 | OR | 0.74 | 0.4 | 1.1 | 0.13 |  |
| 23 | Kantele | 2015 | older age and no travelers’ diarrhea compared to older age and diarrhea | excl. not possible to group | PF | 430 | 90 | 340 | OR | 0.5 | 0.2 | 0.9 | 0.02 |  |
| 24 | Kantele | 2015 | older age (analyzed as continuous variable) | 8. Age | RF | 430 | 90 | 340 | OR | 2.5 | 1.4 | 4.6 | <0.01 |  |
| 25 | Angelin | 2015 | Travel to the south-east Asia region | 3. Travel to southern-Asia | RF | 99 | 35 | 64 | OR | 30 | 6.3 | 147.2 | NA |  |
| 26 | Angelin | 2015 | gender | 7. Gender | NS | 99 | 35 | 64 | OR | 0.6 | 0.1 | 2.7 | NA |  |
| 27 | Angelin | 2015 | older age (analyzed as continuous variable) | 8. Age | NS | 99 | 35 | 64 | OR | 1 | 0.9 | 1.2 | NA |  |
| 28 | Angelin | 2015 | travel length | 6. Length of stay | NS | 99 | 35 | 64 | OR | 1 | 0.98 | 1 | NA |  |
| 29 | Angelin | 2015 | time between return and sampling | excl. not possible to group | NS | 99 | 35 | 64 | OR | 1 | 0.99 | 1.1 | NA |  |
| 30 | Angelin | 2015 | travelers’ diarrhoea | 1. Travelers’ diarrhoea | NS | 99 | 35 | 64 | OR | 0.8 | 0.2 | 3.1 | NA |  |
| 31 | Angelin | 2015 | patient-related healthcare work | excl. not possible to group | NS | 99 | 35 | 64 | OR | 2 | 0.4 | 8.3 | NA |  |
| 32 | Angelin | 2015 | brought disposable gloves | 4. Behavior during travel | NS | 99 | 35 | 64 | OR | 0.3 | 0.1 | 1.4 | NA |  |
| 33 | Angelin | 2015 | strictly consumed bottled water | 4. Behavior during travel | NS | 99 | 35 | 64 | OR | 5 | 0.2 | 128.4 | NA |  |
| 34 | Angelin | 2015 | meticulous hand hygiene | 4. Behavior during travel | NS | 99 | 35 | 64 | OR | 0.8 | 0.1 | 7.7 | NA |  |
| 35 | Angelin | 2015 | antibiotic treatment during travel | 2. Antibiotic use during travel | RF | 99 | 35 | 64 | OR | 5 | 1.1 | 26.2 | NA |  |
| 36 | Paltansing | 2013 | Travel to East Asia | excl. not possible to group | RF | 338 | 113 | 225 | OR | 3.95 | 1.78 | 8.73 | 0.001 |  |
| 37 | Paltansing | 2013 | travel to the middle east | excl. not possible to group | NS | 338 | 113 | 225 | OR | 0.28 | 0.06 | 1.3 | 0.103 |  |
| 38 | Paltansing | 2013 | AB use during travel | 2. Antibiotic use during travel | NS | 338 | 113 | 225 | OR | 1.98 | 0.72 | 5.47 | 0.16 |  |
| 39 | Paltansing | 2013 | median duration of stay | 6. Length of stay | NS | 338 | 113 | 225 | OR | 1 | 0.97 | 1 | 0.22 |  |
| 40 | Paltansing | 2013 | Travel to South Asia | 3. Travel to southern-Asia | RF | 338 | 113 | 225 | OR | 5.09 | 2 | 12.92 | 0.001 |  |
| 41 | Paltansing | 2013 | Travel to Southern Africa | excl. not possible to group | PF | 338 | 113 | 225 | OR | 0.24 | 0.07 | 0.85 | 0.027 |  |
| 42 | Paltansing | 2013 | Travel to South America | excl. not possible to group | PF | 338 | 113 | 225 | OR | 0.14 | 0.03 | 0.59 | 0.008 |  |
| 43 | Ruppé | 2015 | Family visit compared to all inclusive | excl. not possible to group | NS | 574 | 292 | 282 | OR | 1.95 | 0.76 | 4.98 | NA |  |
| 44 | Ruppé | 2015 | backpacking compared to all inclusive | excl. not possible to group | NS | 574 | 292 | 282 | OR | 2.42 | 0.95 | 6.15 | NA |  |
| 45 | Ruppé | 2015 | mix of all-inclusive and tours compared to all inclusive | excl. not possible to group | NS | 574 | 292 | 282 | OR | 1.23 | 0.45 | 3.36 | NA |  |
| 46 | Ruppé | 2015 | B-lactam use during travel | 2. Antibiotic use during travel | RF | 574 | 292 | 282 | OR | 4.08 | 1.39 | 11.97 | 0.011 |  |
| 47 | Ruppé | 2015 | Diarrhoea during journey | 1. Travelers’ diarrhoea | RF | 568 | 291 | 277 | OR | 1.9 | 1.31 | 2.75 | <0.001 |  |
| 48 | Ruppé | 2015 | Organized tour compared to all inclusive | excl. not possible to group | RF | 574 | 292 | 282 | OR | 2.74 | 1.07 | 7.06 | NA |  |
| 49 | Ruppé | 2015 | Travel to Asia compared to Latin America | excl. comparison | RF | 574 | 292 | 282 | OR | 5.72 | 3.55 | 9.24 | NA |  |
| 50 | Ruppé | 2015 | Travel to sub-Saharan Africa compared to Latin America | excl. comparison | RF | 574 | 292 | 282 | OR | 2.21 | 1.4 | 3.48 | NA |  |
| 51 | Kuenzli | 2014 | Travel to Sri Lanka versus India | excl. comparison | PF | 170 | 118 | 52 | OR | 0.05 | 0.02 | 0.16 | <0.001 |  |
| 52 | Kuenzli | 2014 | travel to Bhutan versus India | excl. comparison | NS | 170 | 118 | 52 | OR | 0.66 | 0.13 | 3.3 | 0.651 |  |
| 53 | Kuenzli | 2014 | travel to Nepal versus India | excl. comparison | NS | 170 | 118 | 52 | OR | 0.57 | 0.17 | 1.88 | 0.355 |  |
| 54 | Kuenzli | 2014 | travel reason business versus tourist | excl. not possible to group | NS | 170 | 118 | 52 | OR | 1.58 | 0.44 | 5.71 | 0.483 |  |
| 55 | Kuenzli | 2014 | Travel reason visiting friends and relatives versus tourist | excl. not possible to group | RF | 170 | 118 | 52 | OR | 3.86 | 1.02 | 14.59 | 0.046 |  |
| 56 | Kuenzli | 2014 | Tap water consumption | 4. Behavior during travel | PF | 170 | 118 | 52 | OR | 0.27 | 0.08 | 0.87 | 0.029 |  |
| 57 | Kuenzli | 2014 | Ice Cream and Pastry consumption | 5. Food consumption during travel | RF | 170 | 118 | 52 | OR | 3.9 | 1.61 | 9.43 | 0.002 |  |
| 58 | Kuenzli | 2014 | Length of journey (per week) | 6. Length of stay | RF | 170 | 118 | 52 | OR | 2.08 | ND | ND | 0.01 |  |
| 59 | Vading | 2016 | Male | 7. Gender | NS | 172 | 55 | 117 | OR | 2.11 | 0.96 | 4.65 | 0.063 |  |
| 60 | Vading | 2016 | older age (analyzed as continuous variable) | 8. Age | NS | 172 | 55 | 117 | OR | 1 | 0.98 | 1.03 | 0.8 |  |
| 61 | Vading | 2016 | Indian subcontinent compared to south-east Asia | excl. comparison | RF | 172 | 55 | 117 | OR | 5.62 | 2.27 | 13.89 | <0.001 |  |
| 62 | Vading | 2016 | Northern Africa compared to South-East Asia | excl. comparison | RF | 172 | 55 | 117 | OR | 5.5 | 1.78 | 16.94 | 0.003 |  |
| 63 | Vading | 2016 | Turkey compared to South-East Asia | excl. comparison | NS | 172 | 55 | 117 | OR | 0.81 | 0.15 | 4.32 | 0.81 |  |
| 64 | Vading | 2016 | Travelers’ diarrhoea | 1. Travelers’ diarrhoea | RF | 172 | 55 | 117 | OR | 2.5 | 1.04 | 6.03 | 0.04 |  |
| 65 | Vading | 2016 | Antibiotics during travel | 2. Antibiotic use during travel | RF | 172 | 55 | 117 | OR | 5.92 | 1.27 | 27.2 | 0.024 |  |
| 66 | Vading | 2016 | Chronic disease | excl. not possible to group | PF | 172 | 55 | 117 | OR | 0.27 | 0.1 | 0.76 | 0.014 |  |
| 67 | Reuland | 2016 | Age per 10 year increase | 8. Age | NS | 418 | 98 | 320 | OR | 1.02 | 1 | 1.04 | NA |  |
| 68 | Reuland | 2016 | travelers’ diarrhoea without AB use compared to no TD no AB | 1. Travelers’ diarrhoea | NS | 418 | 98 | 320 | OR | 1.65 | 0.97 | 2.82 | NA |  |
| 69 | Reuland | 2016 | no diarrhoea with AB use compared to no TD no AB | 2. Antibiotic use during travel | NS | 418 | 98 | 320 | OR | 1.49 | 0.24 | 9.08 | NA |  |
| 70 | Reuland | 2016 | travelers’ diarrhoea with AB use compared to no TD no AB | 1. Travelers’ diarrhoea | RF | 418 | 98 | 320 | OR | 9.56 | 2.64 | 34.57 | NA |  |
| 71 | Reuland | 2016 | Travel to only Latin America/Caribbean compared to only Africa | excl. comparison | NS | 418 | 98 | 320 | OR | 1.36 | 0.43 | 4.31 | NA |  |
| 72 | Reuland | 2016 | Travel to only Asia compared to only Africa | excl. comparison | RF | 418 | 98 | 320 | OR | 7.31 | 3.03 | 17.63 | NA |  |
| 73 | Arcilla | 2017 | Occasional meals at street food stalls | 5. Food consumption during travel | RF | 1844 | 631 | 1213 | OR | 1.33 | 1.04 | 1.71 | 0.022 |  |
| 74 | Arcilla | 2017 | Daily meals at street food stalls | 5. Food consumption during travel | RF | 1844 | 631 | 1213 | OR | 1.78 | 1.07 | 2.95 | 0.025 |  |
| 75 | Arcilla | 2017 | Antibiotic use during travel | 2. Antibiotic use during travel | RF | 1829 | 626 | 1203 | OR | 2.69 | 1.79 | 4.05 | <0.001 |  |
| 76 | Arcilla | 2017 | Diarrhoea during travel compared to no diarrhoea | 1. Travelers’ diarrhoea | RF | 1806 | 622 | 1184 | OR | 1.42 | 1.12 | 1.80 | 0.003 |  |
| 77 | Arcilla | 2017 | Diarrhoea during and immediately after travel compared to no diarrhoea | excl. only during travel | RF | 1806 | 622 | 1184 | OR | 2.31 | 1.42 | 3.76 | 0.001 |  |
| 78 | Arcilla | 2017 | Pre-existing bowel disease | excl. not possible to group | RF | 1843 | 630 | 1213 | OR | 2.10 | 1.13 | 3.90 | 0.019 |  |
| 79 | Arcilla | 2017 | Quinolone use during travel | 2. Antibiotic use during travel | RF | 1847 | 633 | 1214 | OR | 6.0 | 2.90 | 12.40 | NA |  |
| 80 | Arcilla | 2017 | Hand hygiene before meals: clean with soap | 4. Behavior during travel | PF | 1844 | 631 | 1213 | OR | 0.77 | 0.60 | 0.99 | 0.044 |  |
| 81 | Arcilla | 2017 | Hand hygiene before meals: clean with alcohol | 4. Behavior during travel | NS | 1844 | 631 | 1213 | OR | 0.97 | 0.66 | 1.44 | 0.885 |  |
| 82 | Arcilla | 2017 | Hand hygiene before meals: clean with alcohol and soap | 4. Behavior during travel | NS | 1844 | 631 | 1213 | OR | 1.12 | 0.79 | 1.59 | 0.518 |  |
| 83 | Arcilla | 2017 | Diarrhoea immediately after travel compared to no diarrhoea | excl. only during travel | NS | 1806 | 622 | 1184 | OR | 1.3 | 0.63 | 2.68 | 0.477 |  |
| 84 | Arcilla | 2017 | Attendance of large (religious) gathering | excl. not possible to group | PF | 1844 | 631 | 1213 | OR | 0.57 | 0.34 | 0.94 | 0.028 |  |
| 85 | Arcilla | 2017 | Beach holiday | excl. not possible to group | PF | 1845 | 631 | 1214 | OR | 0.73 | 0.56 | 0.95 | 0.021 |  |
| 86 | Peirano | 2017 | Visited India | 3. Travel to southern-Asia | RF | 109 | 70 | 39 | OR | 19.9 | 4.5 | 88.8 | NA |  |
| 87 | Peirano | 2017 | Consumed meals with locals | 5. Food consumption during travel | RF | 109 | 70 | 39 | OR | 6.9 | 1.2 | 39.6 | NA |  |
| 88 | Peirano | 2017 | Non-business travel | excl. not possible to group | RF | 109 | 70 | 39 | OR | 12.4 | 2.8 | 55.2 | NA |  |
| 89 | Peirano | 2017 | Use of any antibiotics during travel | 2. Antibiotic use during travel | RF | 109 | 70 | 39 | OR | 4.3 | 1.3 | 14.3 | NA |  |
| 90 | Leangapichart | 2017 | Shortness of breath | excl. not possible to group | RF | 206 | 71 | 135 | OR | 2.56 | 1.14 | 5.73 | 0.023 |  |
| 91 | Leangapichart | 2017 | diarrhoea | 1. Travelers’ diarrhoea | RF | 140 | 47 | 93 | OR | 3.35 | 1.20 | 9.36 | 0.021 |  |
| 92 | Leangapichart | 2017 | Use of macrolides | 2. Antibiotic use during travel | PF | 206 | 71 | 135 | OR | 0.15 | 0.04 | 0.57 | 0.005 |  |
| 93 | Leangapichart | 2017 | at least one chronic disease | excl. not possible to group | NS | 206 | 71 | 135 | OR | ND | ND | ND | 0.076 |  |
| 94 | Leangapichart | 2017 | B-lactam use during travel | 2. Antibiotic use during travel | NS | 206 | 71 | 135 | OR | ND | ND | ND | 0.633 |  |
| 95 | Leangapichart | 2017 | Geographical origin Algeria | excl. not possible to group | NS | 206 | 71 | 135 | OR | ND | ND | ND | 0.534 |  |
| 96 | Leangapichart | 2017 | Geographical origin Morocco | excl. not possible to group | NS | 206 | 71 | 135 | OR | ND | ND | ND | 0.151 |  |
| 97 | Schaumburg | 2019 | Vegetarian diet | 5. Food consumption during travel | PF | 132 | 90 | 42 | OR | 0.4 | 0.2 | 1 | 0.04 |  |
| 98 | Schaumburg | 2019 | Nicotine abuse | 4. Behavior during travel | NS | 132 | 90 | 42 | OR |  | 2.8 | 0.5 | 15.4 | 0.2 |
| 99 | Schaumburg | 2019 | Treatment of diarrhoea during travel | 2. Antibiotic use during travel | RF | 132 | 90 | 42 | OR |  | 3.1 | 1.1 | 9.1 | 0.04 |
| 100 | Schaumburg | 2019 | Hospital admission in the past 12 months | excl. not possible to group | NS | 132 | 90 | 42 | OR |  | ∞ | NA | NA | 1 |
| 101 | Schaumburg | 2019 | Travel to Asia | excl. not possible to group | NS | 132 | 90 | 42 | OR |  | 1.7 | 0.7 | 4.2 | 0.3 |
| 102 | Schaumburg | 2019 | Travel to South Africa | excl. not possible to group | NS | 132 | 90 | 42 | OR |  | 1.3 | 0.3 | 5.5 | 0.7 |
| 103 | Schaumburg | 2019 | Backpacking | excl. not possible to group | NS | 132 | 90 | 42 | OR |  | 1.2 | 0.4 | 3.3 | 0.9 |
| 104 | Schaumburg | 2019 | Visiting friends and relatives | excl. not possible to group | PF | 132 | 90 | 42 | OR |  | 0.3 | 0.1 | 0.7 | 0.009 |
| 105 | Schaumburg | 2019 | Non-bloody diarrhoea | 1. Travelers’ diarrhoea | NS | 132 | 90 | 42 | OR |  | 1.2 | 0.5 | 3.2 | 0.7 |
| 106 | Schaumburg | 2019 | Persisting diarrhoea after the return | excl. not possible to group | NS | 132 | 90 | 42 | OR |  | 1.8 | 0.3 | 10.2 | 0.5 |
| 107 | Schaumburg | 2019 | Vomiting | excl. not possible to group | NS | 132 | 90 | 42 | OR |  | 3.5 | 0.4 | 32.8 | 0.3 |
| 108 | Schaumburg | 2019 | Duration of travel, in mean days | excl. not possible to group | NS | 132 | 90 | 42 | OR |  | 1 | 0.9 | 1 | 0.2 |
| 109 | Schaumburg | 2019 | Frequency of swimming during travel, in median times | 4. Behavior during travel | NS | 132 | 90 | 42 | OR |  | 0.9 | 0.8 | 1 | 0.1 |

Abbreviations: ND, no data; excl., excluded from meta-analyses; RF, risk factor; PF, protective factor; NS, non-significant factor; OR, odds ratio
