# Supplementary material for: Acquisition of multidrug-resistant Enterobacterales during international travel: a systematic review of clinical and microbiological characteristics and meta-analyses of risk factors
Source: Antimicrob Resist Infect Control. 2020 May 20;9:71. doi: 10.1186/s13756-020-00733-6 (PMC7237615; doi:10.1186/s13756-020-00733-6)

**Additional file 5. Risk of publication bias – funnel plots**

1. **Travellers’ diarrhoea**

Begg-Mazumdar: Kendall's tau = 0.5 P= 0.0752 (low power)

Egger: bias = 1.802728 (95% CI = 0.278326 to 3.32713) P= **0.0267**

Funnel plot:


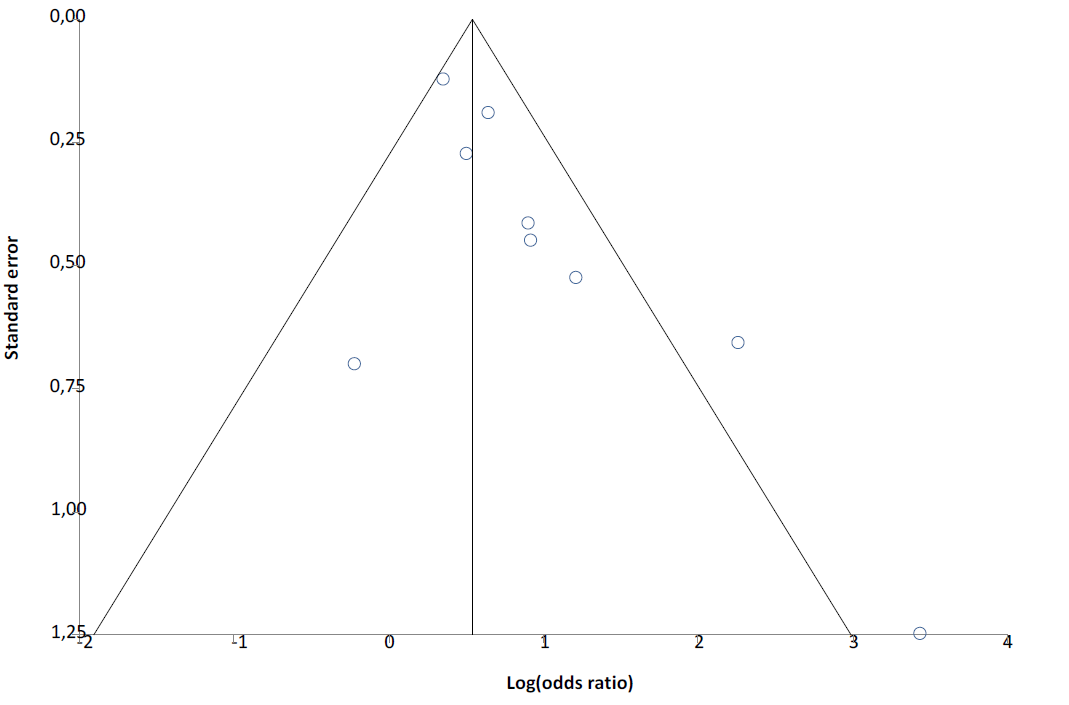


1. **Antibiotic use during travel**

Begg-Mazumdar: Kendall's tau = 0.054545 P= 0.8793

Egger: bias = -0.379707 (95% CI = -2.908047 to 2.148632) P= 0.7419

Funnel plot:


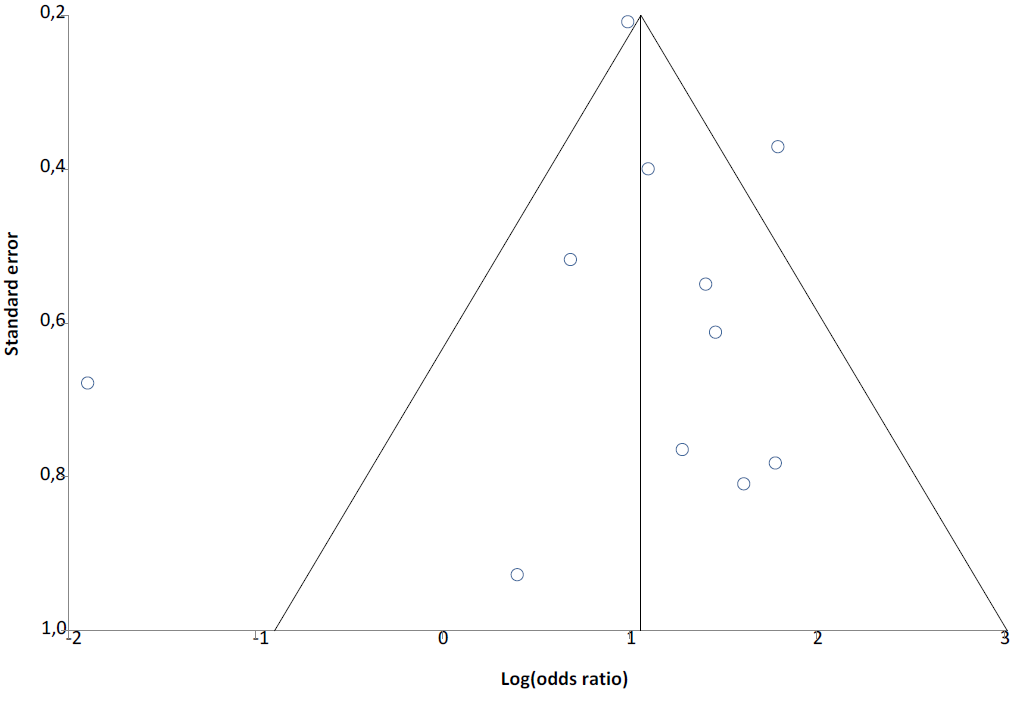


1. **Travel to Southern-Asia**

Begg-Mazumdar: Kendall's tau = 0.666667 P= 0.3333 (low power)

Egger: bias = 4.958949 (95% CI = 3.495597 to 6.422301) P= **0.0047**

Funnel plot:


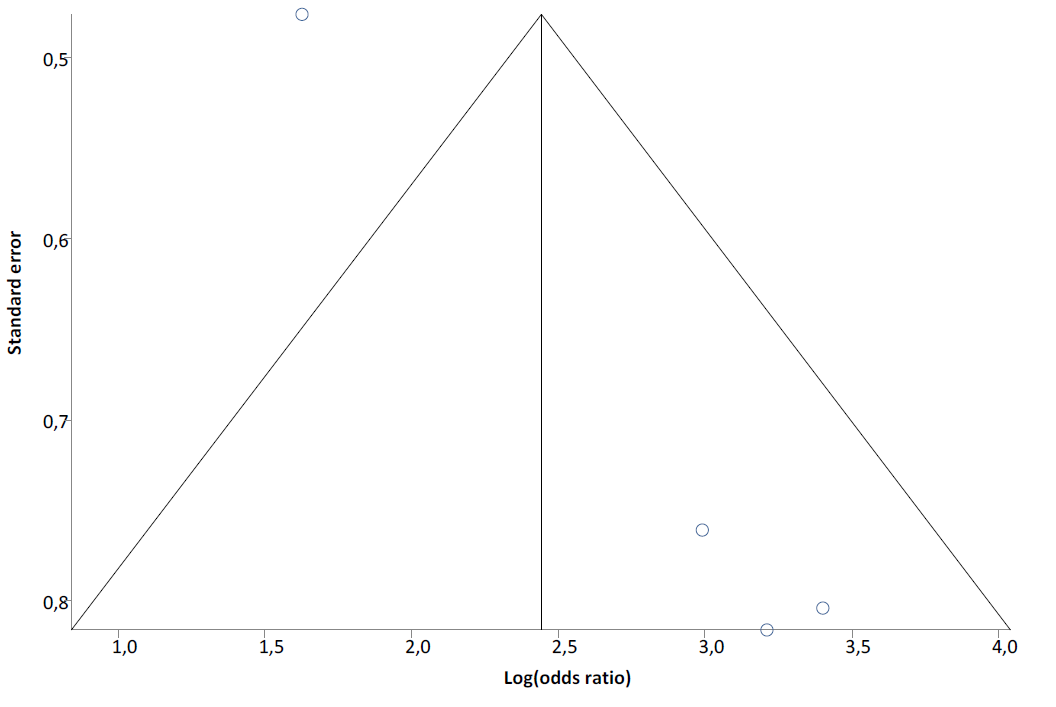


1. **Behaviour during travel**

Begg-Mazumdar: Kendall's tau = 0.047619 P> 0.9999 (low power)

Egger: bias = -0.440636 (95% CI = -2.652375 to 1.771102) P= 0.6304

Funnel plot:


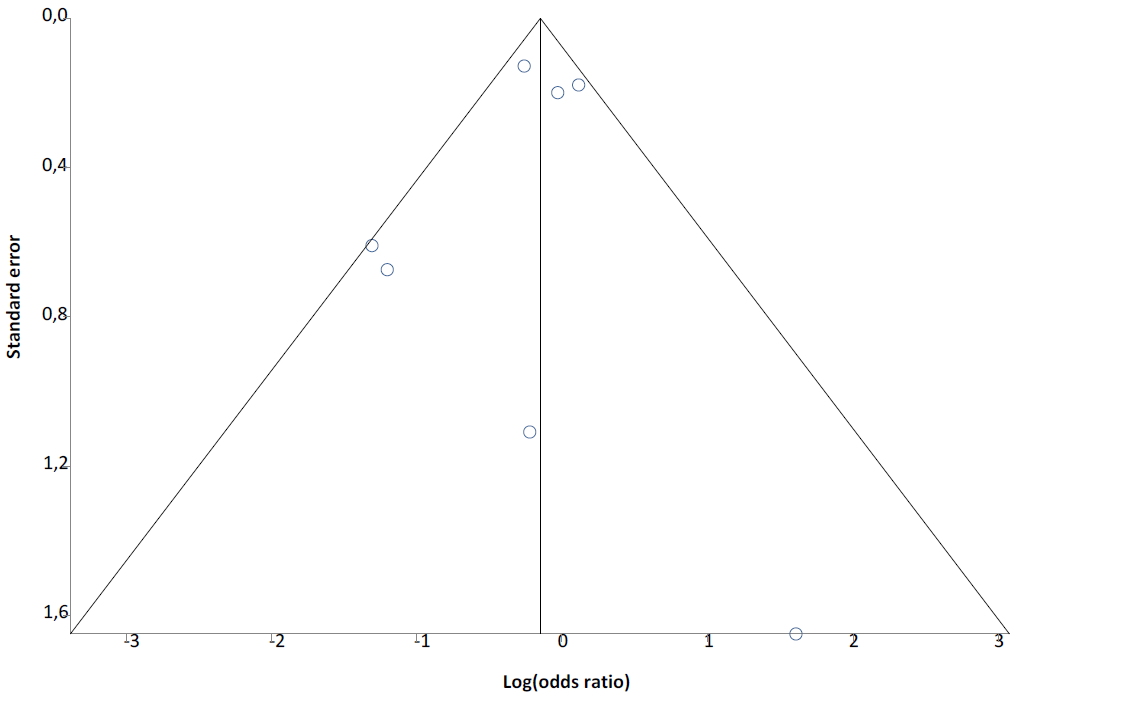


1. **Food consumption**

Begg-Mazumdar: Kendall's tau = 0.2 P= 0.8167 (low power)

Egger: bias = 0.770067 (95% CI = -5.032782 to 6.572916) P= 0.7012

Funnel plot:


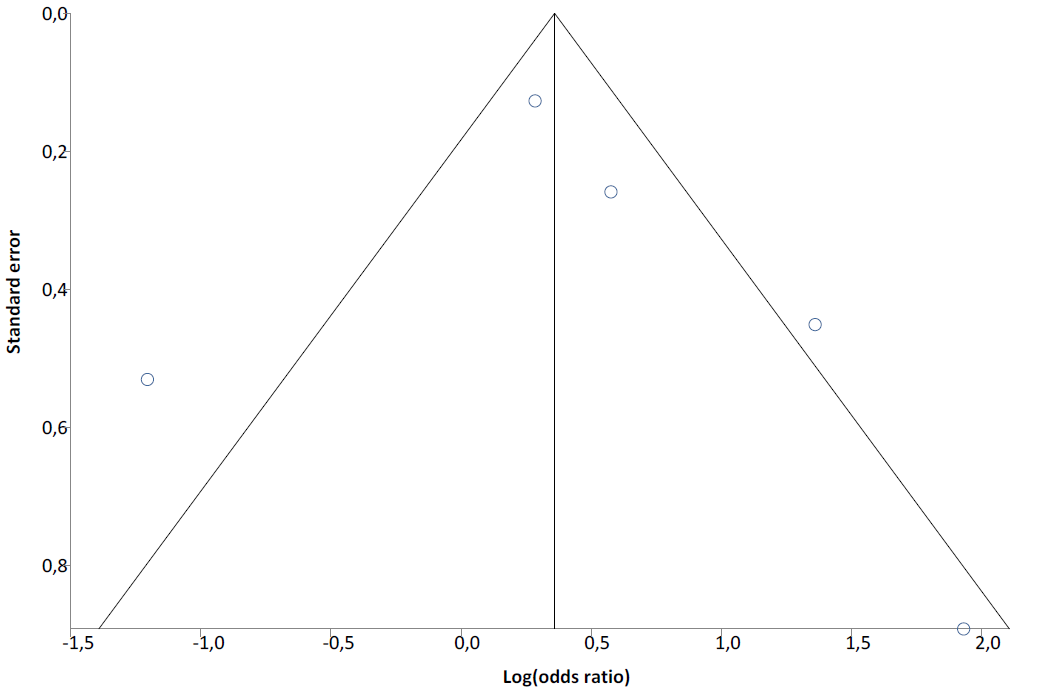


1. **Sex**

Begg-Mazumdar: Kendall's; too few strata

Egger: bias = too few strata

Funnel plot: too few strata

1. **Older age**

Begg-Mazumdar: Kendall's tau = 0.52381 P= 0.1361 (low power)

Egger: bias = 2.086117 (95% CI = 0.599575 to 3.572658) P= **0.0154**

Funnel plot:


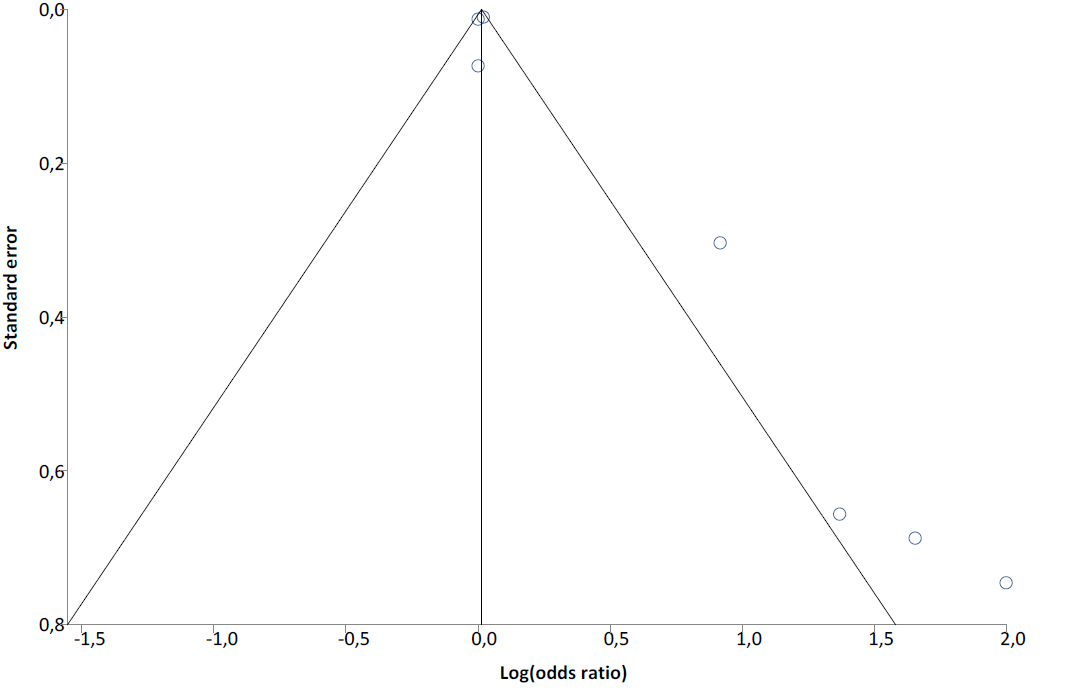

Supplement: Supplementary file 5 — Additional file 5:. text file: Risk of publication bias – funnel plots. [file 13756_2020_733_MOESM5_ESM.docx]
